# The use of drone-delivered Automated External Defibrillators in the emergency response for out-of-hospital cardiac arrest. A simulation study

**DOI:** 10.1016/j.resplu.2025.101045

**Published:** 2025-07-25

**Authors:** Christopher M. Smith, Carl Powell, Celia J. Bernstein, Harry Howe, Mark Holt, Mary O’Sullivan, Keith Couper, Nigel Rees

**Affiliations:** a Warwick Clinical Trials Unit, University of Warwick, Coventry CV4 7AL, UK; b Welsh Ambulance Services University NHS Trust, Pre-Hospital Emergency Research Unit (PERU), Institute of Life Sciences Swansea University Singleton Park, Swansea, Wales, UK; c SkyBound Rescuer, Unit 1 Keel Rd, Woolston, Southampton SO19 9UY, United Kingdom; d Patient and Public Involvement (PPI) representative, United Kingdom; e Critical Care Unit, University Hospitals Birmingham NHS Foundation Trust, Birmingham, UK

**Keywords:** Out-of-hospital cardiac arrest, Cardiopulmonary resuscitation, Defibrillation, Public access defibrillation, Drones, Unmanned aerial devices, Emergency medical services

## Abstract

**Background:**

Drones are now being used to deliver Automated External Defibrillators (AEDs) for out-of-hospital cardiac arrest. Delays occur before (between emergency call and drone take-off) and after drone flight itself (related to bystander interaction with drone/AED). The emergency call-handler may have an important role in helping bystanders retrieve and use an AED.

**Methods:**

Following an emergency (999) call for simulated out-of-hospital cardiac arrests, a remote drone was activated and made autonomous Beyond Visual Line of Sight flights to the scene. Real-time communications between drone operator and call-handler allowed participants to receive updates about drone progress. Outcomes included hands-off CPR time, time away from patient’s side retrieving the AED, time from emergency call to start of drone flight, and time from drone arrival to AED shock. We used video-recording, emergency-call audio and post-simulation interviews to gather additional information about participants’ experiences.

**Results:**

We conducted 11 single bystander simulations and successfully delivered an AED on 9 occasions. It took (median) 2:18 min (interquartile range, IQR 2:13–2:38 min) from emergency call to drone take-off, and a further 4:35 min (3:39–4:46 min) once the drone had arrived on scene until first shock. Hands-off CPR time was 2:32 min (2:01–2:46 min); 0:16 min (0:13–0:21 min) of this was spent retrieving the AED. Bystanders retrieved the AED safely and interacted well with the drone, but often struggled with AED use.

**Conclusion:**

We demonstrated effective real-time communication during simulations. Drone start-up procedures were quick but there were delays once the drone arrived on scene. Bystanders and call-handlers need more support to effectively use drone-delivered AEDs.

## Introduction

Around 1 in 10 people who have an out-of-hospital cardiac arrest (OHCA) will survive to hospital discharge or to 30 days.^1^, ^2^, ^3^ Prompt bystander cardiopulmonary resuscitation (CPR) and Automated External Defibrillator (AED) use can substantially increase survival rates.^4^

Bystander defibrillation using AEDs occurs in approximately 5–10 % of OHCAs,^3^, ^5^ but is associated with a doubling in the odds of survival with favourable neurological outcomes.^6^ Many barriers to bystander AED use relate to a bystander’s ability to promptly locate, access and retrieve a publicly-available AED. A 2017 systematic review of barriers to AED use reported that only 5–22 % of people knew the location of their nearest AED, 18–59 % of AEDs were poorly accessible and only 3–25 % of OHCAs occurred within 100 m of a publicly-available AED.^5^

One way of overcoming these barriers may be to deliver AEDs using flying drones. Simulation studies have modelled optimal drone locations^7^, ^8^, ^9^ and demonstrate that a drone-delivered AED could have arrived at historical OHCAs much quicker than with a standard ambulance service response.^7^, ^8^, ^10^, ^11^, ^12^ User experience of drone interaction during OHCA simulations is generally positive,^13^, ^14^, ^15^ with untrained bystanders reporting more concerns about using AEDs than interacting with the drones.^15^ Early studies describing the implementation of drone-delivered AEDs in Sweden^16^ and Denmark^17^ have described successful AED delivery in a small number of cases.

The optimum system for drone-delivery of AEDs is currently unknown. Our previous simulation study demonstrated the feasibility of using a drone to carry an AED, the ease with which participants interacted with the drone, and the need for call-handler support during the emergency (‘999′) call made during the simulation.^18^ In this current study we aimed to establish Beyond Visual Line of Sight (BVLOS) drone flight for AED delivery for the first time, demonstrate effective real-time communication between drone operator, call-handler and bystander and identify the main issues affecting bystander use of drone-delivered AEDs.

## Methods

**Fig. 1:**
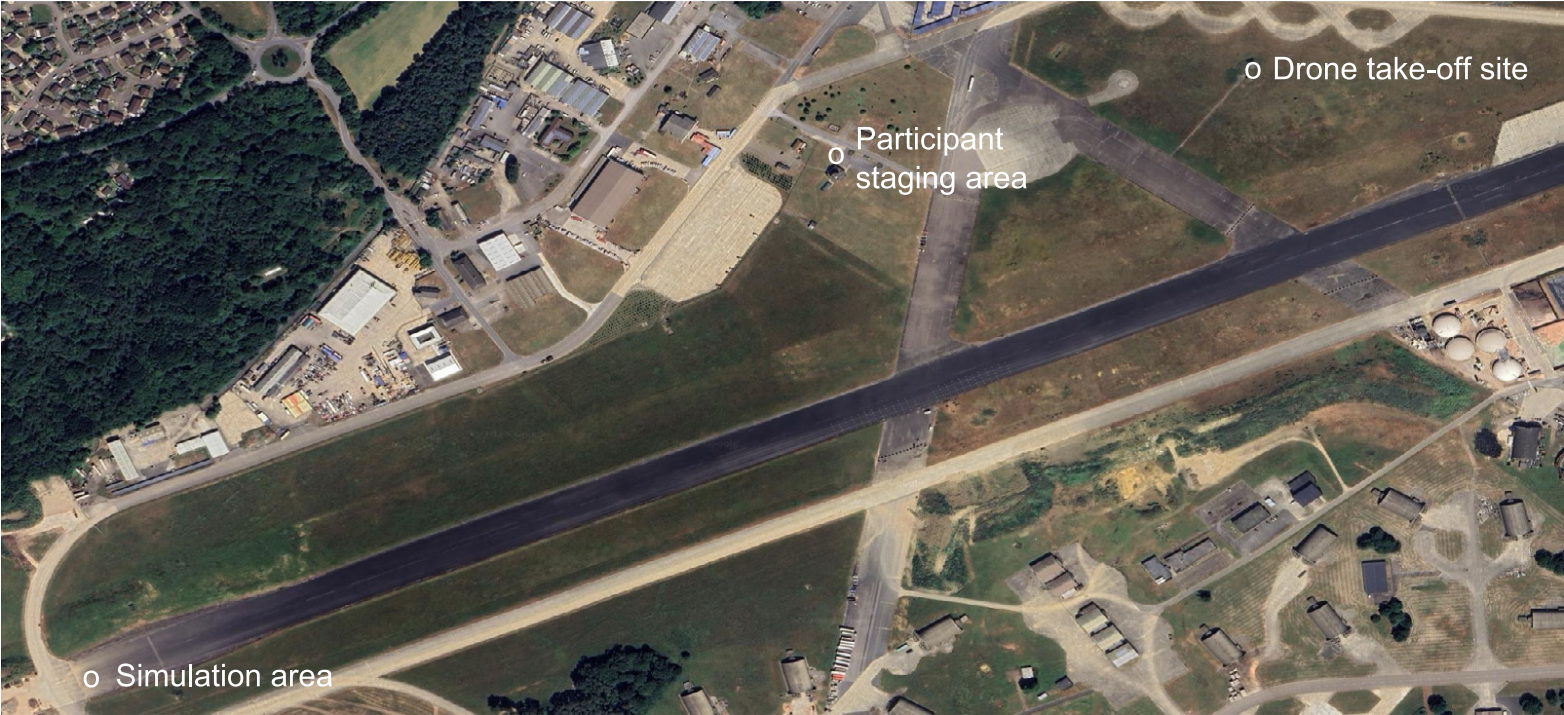
Study site overview.

We conducted a mixed-methods simulation study, recording key study timings as well as conducting surveys, interviews and audiovisual review of simulations to provide a detailed summary of the participants’ views of and their behaviours and interactions with drone, AED and the call-handler. We conducted OHCA simulations in September 2024 at a disused airfield in the UK (see Fig. 1).

### Recruitment

We identified local businesses and groups by internet searches, and requested that they share study information with members of their organisation. We included adults who reported that they were able to use a mobile phone and had the physical and mental capability to perform CPR and use an AED in an outdoor environment. Individuals who were pregnant or considered themselves to be unable to complete the simulation because of physical or psychological distress were excluded. Participants provided written informed consent.

In our previous simulation study we recruited 18 participants without difficulty and gathered a wealth of information about how processes surrounding AED delivery by drone might be optimised.^18^ Given this and the daily maximum limit on flights, we set an a priori sample size of 20 participants in this study.

### Study conduct

Drone flight and all flight-safety requirements were overseen by personnel from SkyBound Rescuer (https://skyboundrescuerproject.com/), a UK-based commercial company specialising in the use of drones for public safety. SkyBound created an Operational Safety Case for flight with and carriage/delivery of the AED, and the UK Civil Aviation Authority provided necessary approvals for BVLOS drone flight in the study.

**Fig. 2:**
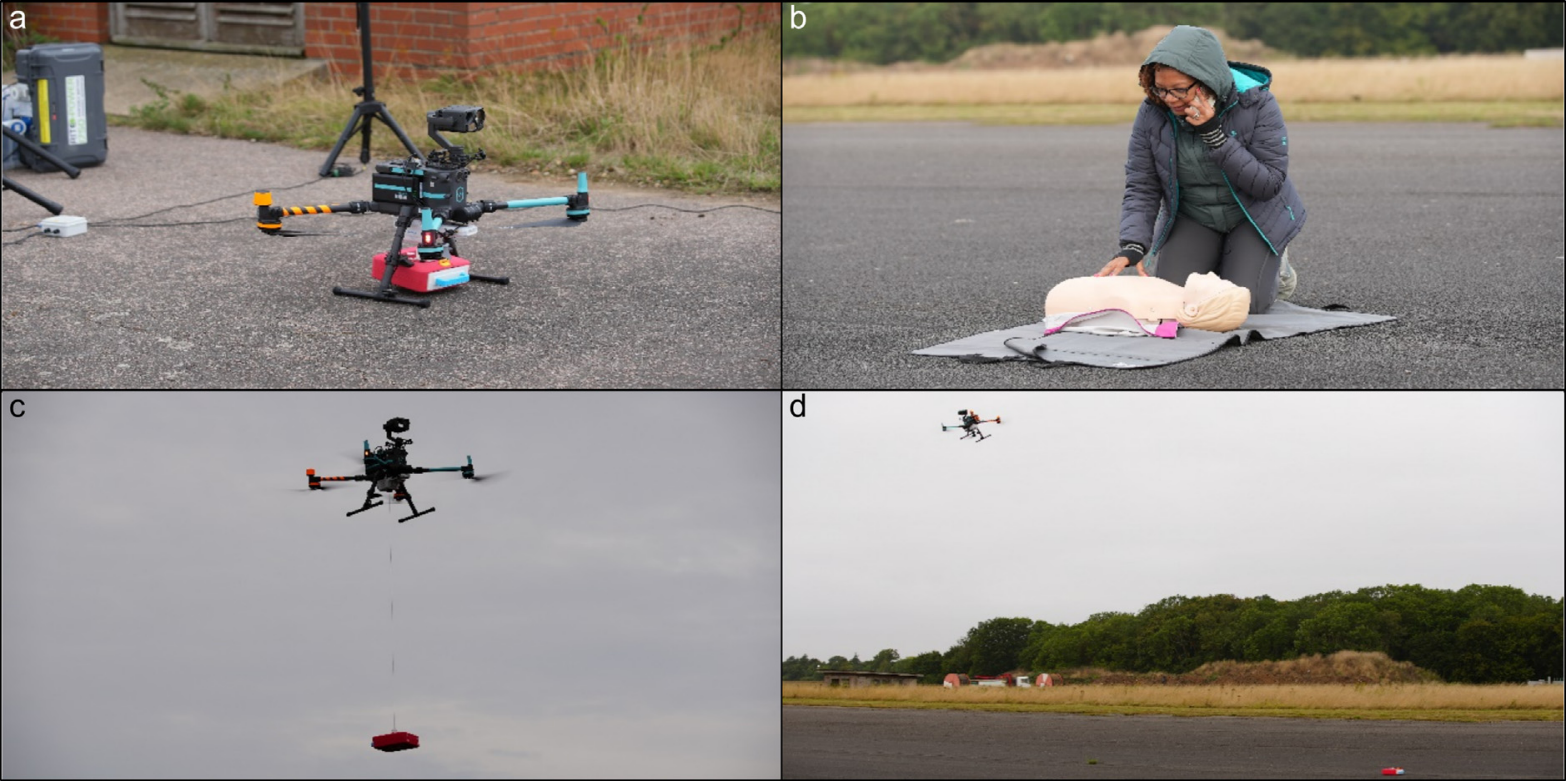
a) drone at remote site with AED loaded, b) emergency call made (study team member shown), c) drone on-scene winching AED to ground, d) drone departs and bystander informed it is safe to retrieve AED on ground.

We used a DJI M300 drone with a custom docking station setup, configured with front- and downward facing camera, winch attachment (Speedip system) designed to hold a custom-built carry case (created by SkyBound) for a Schiller FRED Easyport Plus Training AED. Custom feet were installed to prevent defib sway during flight. A “puck” was also produced by SkyBound to enable a connection to the drone’s onboard power supply and control link to the winch. The drone was stationed outside (not in a hangar) at a remote site (see Fig. 2**)** and was shut off between simulations.

On arrival at the simulation site, we asked participants to present to an indoor staging area, before proceeding to the simulation site itself. The drone pilot was remote to the study site. Drone flight was autonomous, with the pilot able to intervene if required via drone-mounted video and contact with on-scene safety personnel.

The drone pilot had a mobile device provided by Welsh Ambulance Services University NHS Trust (WAST). Once cardiac arrest was identified by the call-handler during the emergency call, the call-handler allocated the cardiac arrest incident to this device (‘incident allocation’). The drone pilot then began start-up procedures, including use of a bespoke mission allocation system (at the remote location where they were situated). Once complete, the drone took-off. Shortly after take-off, once the drone had successfully begun horizontal movement towards the scene, the drone pilot indicated to the call-handler (via push notification using the mobile device) that the drone was mobile. The pilot then indicated to the call-handler when the drone had ‘arrived on scene’ (once it had stopped horizontal flight and was hovering) and when it was ‘safe to approach’ (AED winched to ground, the winch retracted and the drone having initiated its return flight). The drone hovered at 10 m above the ground when winching down the AED, with a 20 m horizontal separation from the participant. At no point was the participant directly underneath the drone. The drone did not have an emergency parachute.

At the simulation site, the participant was told that they were by themselves (i.e. this was a single bystander scenario) and they would find an unconscious patient who was not breathing – a CPR manikin (Laerdal Little Anne). The participant was asked to indicate when they wished to make an emergency call, at which point a member of the research team placed the call to a WAST 999 training centre and immediately handed them the mobile phone. There was no further input from the study team.

The simulation was video−recorded and CMS determined on-scene timings after video review. WAST made emergency call audio logs available to the research team, which allowed us to determine the drone ‘arrived on scene’ and ‘safe to approach’ timings from the start of the emergency call. SkyBound provided the time from incident allocation receipt to time of take-off.

The emergency call was answered by a trained call-handler and they proceeded according to existing protocols, but with additional instructions: when the drone was ‘mobile’ – *“We are sending a defibrillator to your location by flying drone. Please continue CPR, and I will let you know when it arrives and it is safe for you to get the defibrillator.”;* when the drone had arrived on scene – *“Please do not leave the patient yet. The drone has arrived on scene. I will tell you when it is safe to get the defibrillator.”;* when it was ‘safe to approach’ – *“It is now safe to leave the patient. Please get the defibrillator and bring it back to the patient as quickly as you can.”*

The drone lowered the AED to the ground via winch with a quick-release mechanism activated on impact with the ground. The participant retrieved the AED when told it was safe to do so and attached it to the patient. The AED delivered a shock and the simulation ended once the participant restarted CPR. Fig. 2 illustrates the drone, AED and simulation site.

### Outcomes and analyses

We recorded the following timings:

• From emergency call to incident allocation and drone take-off
• Flight distance and time
• From arrival on scene to ‘safe to approach’
• Time away from the patient to retrieve the drone-delivered AED
• Hands-off CPR time (from the time the participant stopped chest compressions to retrieve the AED to starting chest compressions again after the AED delivered a shock)
• From arrival on scene to AED attached and first shock

We reported categorical data as number and percentage, and continuous data as median and interquartile range (IQR). We collected participant feedback after the simulation with a brief (<5 min) interview and a questionnaire probing their experiences of retrieving the AED, based on the ‘System Usability Scale’ (SUS), developed to evaluate usability of new devices or systems.^19^, ^20^ Study timings, interview topic guide and SUS are available in the Electronic Supplementary Material**.**

We reviewed on-scene video and emergency call audio, and obtained feedback from study team members present at the simulation and the call handlers answering the calls. For the qualitative analysis of participant behaviour we took an interpretivist approach,^21^ recognising the complexity and unpredictable nature of the interaction between participants and their environment.^22^ For each participant, CMS conducted the post-event interview, reviewed simulation audio and video, and took field notes informed by observations from the research team. CMS sorted data and, using an iterative approach, produced key themes from the data collected. These were reviewed by CP. These themes and key observations in each theme were synthesised into a table. Original notes and data arranged by themes are available in the Electronic Supplementary Material**.**

### Ethical considerations and reporting

The Health Research Authority and Health and Care Research Wales (REC reference 24/WA/0034) granted ethical approval for the study on 11/03/2024. We prospectively registered the study at ClinicalTrials.gov (NCT06334718). We have reported this article with reference to the STROBE (Strengthening the Reporting of Observational Studies in Epidemiology) guidelines for observational studies,^23^ and its extension for simulations.^24^

### Patient and public involvement

There were two patient and public involvement representatives. They reviewed the project protocol and all participant-facing documents, observed and offered feedback on study days, and co-authored this article.

## Results

We invited 22 participants to participate, but cancelled 8 before their allocated day/time because of adverse weather. Of the 14 that arrived on scene and were consented, 11 participated in an OHCA simulation. We cancelled the simulations of the remaining three participants on scene due to poor weather (n = 2) and technical difficulties (n = 1). In two simulations automatic safety features triggered the drone to ‘Return to Base’ after it arrived on scene following intermittent connectivity issues interfering with the drone’s ability to report its live location, so we achieved AED delivery in nine simulations. In two of these simulations, the participants did not deliver an AED shock – these simulations were ended when participants restarted CPR.

Among the 11 participants, 73 % (n = 8) were male and the median age was 62 (IQR 39–70) years. One participant (9 %) had previous real-world experience of using an AED during an OHCA **(**Table 1**).**

**Table 1:** Participant details (n = 11).

Age in years (median, IQR)			62 (38.5–70)
Gender – male (n, %)		8	(73 %)
Previous training (in last five years) (n, %)	*CPR only* *CPR and AED* *Neither*	3 4 4	(27 %) (36.5 %) (36.5 %)
Previous real-world experience n, %	*CPR only* *CPR and AED* *Neither*	1 1 9	(9 %) (9 %) (82 %)

IQR interquartile range.

### Study timings

From answering the emergency call, it took (median) 1:28 min (IQR 1:23–1:49 min) for the cardiac arrest incident to be allocated to the drone pilot and 2:18 min (2:13–2:38 min) until take-off. Flights (from moment of take-off to stopping / hovering at the scene) were between 1.15 and 1.28 km in distance and lasted 2:19 min (2:15–2:25 min), so that the drone arrived on scene 04:43 min (04:32–04:57 min) after the start of the emergency call. Once the drone arrived on scene, it was a further 1:47 min (1:46–1:51 min) until it was safe for the participant to approach. Arrival on scene to first shock took 4:35 min (3:39–4:46 min). Total hands-off CPR time was 2:32 min (2:01–2:46 min), of which 16sec (13-21sec) was time that the participant spent away from the patient. Total simulation time (999 call to first shock) was 09:13 min (08:11–10:07 min). Table 2 has further details.

**Table 2:** Key simulation day timings (median, IQR).

Flight distance (km)	1.17	(1.16–1.21)	(n = 11)
Emergency call to incident allocation (mm:ss)	01:28	(01:23–01:49)	(n = 11)
Incident allocation to take off (mm:ss)	00:49	(00:48–00:50)	(n = 11)
Emergency call to take off (mm:ss)	02:18	(02:13–02:38)	(n = 11)
Flight time (mm:ss)	02:19	(02:15–02:25)	(n = 11)
Total time emergency call to arrival on scene (mm:ss)	04:43	(04:32–04:57)	(n = 11)
Arrival on scene until to ‘safe to approach’ (mm:ss)	01:47	(01:46–01:51)	(n = 9)
Time away from patient retrieving AED (mm:ss)	00:16	(00:13–00:21)	(n = 9)
Hands-off CPR time (mm:ss)	02:32	(02:01–02:46)	(n = 9)
Arrival on scene to AED attached (mm:ss)	04:10	(03:04–04:34)	(n = 7)
Arrival on scene to first shock (mm:ss)	04:35	(03:39–04:46)	(n = 7)
Total time emergency call to first shock (mm:ss)	09:13	(08:11–10:07)	(n = 7)

The post-simulation questionnaire indicated that participants found it easy to interact with the drone and had variable levels of confidence doing so **(**Table 3**).** Post-simulation interviews, observation and audiovisual review demonstrated the importance of participants’ communication with the call-handler throughout the call. Problems arose when participants found it hard to hear the call-handler (exacerbated by drone noise as it approached), and there were interruptions to CPR when the participant had to listen to and/or clarify call-handler instructions. AED voice instructions and ongoing call-handler instructions would often conflict, or the call-handler’s instructions about AED use were out-of-sync with the AED’s voice instructions.

**Table 3:** Post-simulation questionnaire findings (n = 9).

	**Score (from 1 = strongly disagree to 5 = strongly agree)**				
	**1**	**2**	**3**	**4**	**5**
I found it unnecessarily complex	7	2	0	0	0
I thought it was easy to do	0	2	2	3	2
I felt very confident doing this	2	1	2	1	3
I would imagine that most people would be able to do this	0	2	3	2	2

Participants reacted to the sight and noise of the drone, but it did not stop them performing CPR. All participants obeyed instructions from the call-handler about not approaching the drone prematurely. There were delays to the drone being ‘safe to approach’ after its arrival on scene because of a slow winching mechanism, and participants expressed frustration about the time taken for the AED to reach the ground. There were no hesitations retrieving the AED when asked to do so.

Several participants found it difficult to both remove the AED from its carry case and to use it. There were no device-specific instructions available from the call-handler, and little evidence of the call-handler checking correct AED usage. On two occasions the participant failed to attach the AED. Key themes and observations are detailed in Table 4**.**

**Table 4:** Key themes.

**Theme**	**Sub-theme**	**Observations and participant behaviours**
Communication between call-handler and bystander	*The effect of drone noise on communications*	• Difficulty hearing call handler during drone delivery of AED • Interruptions to CPR to put phone to ear
	*Discussions about retrieving the AED*	• Waiting for 'safe to approach' instruction before leaving patient • Seeking clarification from the call-handler before leaving patient • Providing updates to call-handler when they heard/saw drone
	*Liaising with call-handler to retrieve and use the AED*	• Bystander prioritising one set of instructions (e.g. AED voice instructions) over another (e.g. call handler instructions) • Difficulty hearing instructions when delivered at the same time • Call-handler giving instructions out-of-sync with AED prompts • Waiting for call-handler before starting to access AED
	*Other communication with emergency call handler*	• Listening to call-handler during CPR distracting • Holding phone to ear whilst receiving instructions • Stopping spontaneously initiated CPR once emergency call answered • Clarifying location delayed start of CPR instructions • Clarifying CPR instructions delays CPR initiation
*Communication between call-handler and drone operator*		• No 'abort' instructions available to call-handler • No 'drone delay' instructions available to call handler
Interaction with the drone	*Drone as a distraction*	• Distracted by or looking around in response to drone noise • Updating call-handler when participants could see or hear drone • Call-handler clarifying instructions not to leave patient until told
	*Interacting with drone and retrieving AED*	• No hesitation retrieving AED when instructed • Different approaches to drone – walk vs run; taking phone vs leaving with patient; working out how to open AED casing on way back to patient vs not • Frustration of seeing AED on winch before able to retrieve it • Physical difficulties rising from kneeling position • Desire for the defibrillator to land closer to them
	*Drone delays*	• Delays between drone 'on scene' and AED 'safe to retrieve' perceived as too long by bystanders
Using the AED	*Accessing the AED*	• No device-specific instructions available from call-handler • Difficulties and delays in bystander opening AED casing • AED casing opening mechanism perceived as unusual and difficult to use.
	*Operating the AED*	• Instances of incorrect AED use • Limited intervention by call-handler to check or fix errors in AED use
Bystander wellbeing	*Bystander emotion*	• Extreme participant emotion was difficult for call-handler • Seeing and hearing the drone was reassuring
	*Advice and reassurance from the call-handler*	• Participants sought clarification and reassurance from call-handler • Lots of quiet periods on the emergency call • Use of non-scripted, short, reassuring phrases by call-handler
Participant perceptions of system efficacy		• Physical difficulties performing CPR might limit effectiveness • Belief this would help in rural locations • Lack of belief in capability to use AED

## Discussion

In this study we successfully demonstrated autonomous BVLOS flight of an AED-capable drone to simulated OHCAs. The study adds to the limited evidence base about the use of drone-delivered AED use.^25^ In particular, we have demonstrated the benefits of real-time communications between an emergency call-handler and a remote drone pilot, and the potential difficulties in interactions between call-handlers and bystanders. Time from the start of the emergency call to start of drone flight was (median) 2:18 min and flight time was 2:19 min for a 1.17 km distance. Once the drone reached the scene, it took a further 4:10mins before the AED was attached and 4:35mins before shock delivery. Hands-off CPR time was 2:32 min. Only 16 s of this was time away from the patient retrieving the AED, with much of the rest of the time taken up with attaching and using the AED once the participant had returned to the patient’s side, before restarting CPR.

Participants reported that the task was easy but expressed variable levels of confidence doing it. There were difficulties interacting with the call-handler, and conflicting or out-of-sync call-handler instructions about AED use once the participant had retrieved it. Our use of bystanders with, mostly, little experience of OHCA management and AED use highlighted that helping call-handlers facilitate bystanders in AED use is at least as important as the mode of AED delivery.

The goal is operational drone flight in the UK, which others have demonstrated is possible.^16^, ^17^ Our study demonstrates that to optimise drone-delivery of AEDs there should be a focus on reducing delays before and after drone flight itself, and effective communication between drone operator and call-handler, and between call-handler and bystander. This will help prepare bystanders for the arrival of the drone and – arguably more importantly – to effectively use an AED.

We demonstrated in an earlier simulation involving Visual Line of Sight (VLOS) flight that participants needed reassurance and permission from the call-handler to leave the patient.^18^ In our current study, the call-handler had real-time updates from the drone operator during flight and after arrival, and could safely instruct the participant about AED retrieval. We noted far less hesitation about leaving the simulated patient in the current study.

Our participants found interaction with the drone uncomplicated. This has been noted in other simulation studies,^11^, ^13^, ^14^, ^15^ although there are reports that some participants expressed concerns about retrieving an AED from a landed drone.^11^, ^14^ A simulation study from Sweden involving older adults (median age 71, compared to 62 years in our study) with no recent CPR training similarly found that it was communications with a mobile device and AED use, rather than the drone, that caused most concerns.^15^

There are operational drone-delivery systems in Sweden^16^, ^26^ and Denmark.^17^ These have demonstrated feasible and safe AED delivery, but AEDs seldom get attached to patients.^16^, ^17^ In Sweden (April 2021–May 2022), researchers reported two people shocked by a drone-delivered AED, one of whom survived to 30 days, from a total of 72 drone deployments.^16^ In Aalborg, Denmark, drones were dispatched to 16 eligible OHCAs between June 2022 and April 2023. On no occasion was an AED attached to a patient, although this was due to short ambulance service response times.^17^

Drone-delivered AEDs may be cost-effective if drones are placed in appropriate locations,^27^ although this assumption is based on improved patient survival not yet reported in clinical studies.^28^ There are many potential delays to AED delivery by drone that occur before (from time of emergency call to drone take-off) and after (once arrived on scene before it is safe to retrieve the AED) the drone flight itself.^29^

A scoping review of drone-delivered AEDs reports simulation studies consistently finding a time benefit of over three minutes in AED delivery.^28^ Minimising delays before flight itself (optimising automated drone-activation and take-off procedures, real-time flight planning) and once the drone arrives on scene (optimising winch or landing procedures) will help maximise time benefits. In our study we experienced marked delays after drone arrival because of an unexpected problems with a slow winching mechanism. We have previously used a more rapid winch system^18^ and this, like other technological difficulties, will not be difficult to rectify. We chose to run single bystander scenarios as prolonged time away without performing CPR from the patient may impact outcome^30^ and may not be acceptable to bystanders^31^ and call-handlers.^32^ In this study, there was limited time spent away from the patient retrieving the AED and this may mean that dispatching an AED by drone to single bystanders may be considered acceptable. This is likely to be less of an issue where there are multiple bystanders at the scene willing and able to provide CPR – in one area of the UK (2022–2023) more than one bystander was identified as being at the scene in 64 % (289/451) of 999 calls.^33^

Improving real-time communication between drone operator, emergency call-handler and people at an OHCA is also crucial. People who have been bystanders at OHCAs in the UK reported concerns about leaving patients and whether they would have sufficient capability to locate, retrieve and use a drone-delivered AED. They also felt more anxiety about using an AED than about performing CPR.^31^ Call-handlers in the operational Swedish drone-delivered AED system reported uncertainties about how to guide people in AED use, for how long chest compressions could be paused and what bystanders’ physical and psychological limitations were.^32^

It will be important to determine appropriate clinical parameters for drone dispatch, to examine the robustness of drone-delivery systems, to integrate drone-delivery seamlessly into local EMS systems and to introduce standardised scripts for the call-handler so that they can best support bystanders.^28^ Indeed, although dispatcher-instructions for AED use are widely reported,^34^, ^35^ there is little evidence of their effectiveness and how to optimise them.^36^ Adding a drone may make things more complicated for the bystander, and this could compromise the resuscitation effort if sub-optimally implemented. Other issues, such as weather conditions limiting drone flight, and how to manage technical issues during flight and mission abort parameters because of technical concerns or changing clinical information also need to be considered when establishing drone sites.

Our simulation study has important limitations. First, adverse weather conditions and technical challenges (particularly connectivity issues, whose nature was identified and rectified during the simulation process) meant we were unable to achieve our target sample size. Nevertheless, our mixed-methods study has provided rich and detailed findings that will inform future work, particularly in relation to drone connectivity and its winch mechanism. Second, we used a lightweight training AED, which may not reflect the AED model that would be adopted in clinical practice. Thirdly, the SUS and brief post-simulation interviews are not validated methods of collecting data in cardiac arrest research, although we have previously successfully used this same approach.^18^ We also only recorded if a participant had received CPR or AED training (or both) in the last five years, not the nature of this training or its exact timing. This may feasibly impact upon how effectively the participant interacted with the call-handler. A formal investigation of people’s behaviours during an OHCA when an AED was delivered by drone using validated frameworks (e.g. the Behaviour Change Wheel^37^) could help in the design of interventions to optimise the system. Fourthly, although we demonstrated effective real-time communications and post-AED delivery delays in this simulation there are other aspects of drone integration into the emergency response that we did not simulate. These include not using a hangar to store the drone when not in use, not simulating delays for air traffic control permissions for unscheduled drone flight, and not addressing mechanisms for post-event AED retrieval and analysis.

## Conclusion

We have demonstrated effective real-time communication between emergency call-handlers and a remote drone operator in this simulation study, but there were notable delays once the drone arrived on the scene before the AED was safe to use. Call-handlers followed instructions to safely retrieve the AED without concern, but there were difficulties in using the AED once it had been retrieved. There is great opportunity to improve the interaction between call-handler and bystander to facilitate timely and effective use of the AED once the drone has delivered it to the scene.

## CRediT authorship contribution statement

**Christopher M. Smith:** Writing – review & editing, Writing – original draft, Supervision, Resources, Project administration, Methodology, Investigation, Funding acquisition, Formal analysis, Data curation, Conceptualization. **Carl Powell:** Writing – review & editing, Validation, Methodology, Investigation. **Celia J. Bernstein:** Writing – review & editing, Project administration, Methodology. **Harry Howe:** Writing – review & editing, Software, Resources, Investigation. **Mark Holt:** Writing – review & editing, Investigation. **Mary O’Sullivan:** Writing – review & editing, Investigation. **Keith Couper:** Writing – review & editing, Methodology. **Nigel Rees:** Writing – review & editing, Supervision, Methodology.

## Funding

This project was funded by the 10.13039/501100000272National Institute for Health and Care Research (NIHR) Research for Patient Benefit programme (Grant Reference Number NIHR204382) and 10.13039/100012068Health and Care Research Wales (IRAS: 318417). The views expressed are those of the author(s) and not necessarily those of the NIHR, the Department of Health & Social Care or Health and Care Research Wales.

## Declaration of competing interest

Keith Couper is an Editorial Board Member/Editor-in-Chief/Associate Editor/Guest Editor for this journal and was not involved in the editorial review or the decision to publish this article.

The authors declare the following financial interests/personal relationships which may be considered as potential competing interests: Christopher M Smith has volunteer roles with Resuscitation Council UK, European Resuscitation Council and the International Liaison Committee on Resuscitation.

Nigel Rees receives R&D funding from Health & Care Research Wales who also provided NHS delivery funding for this project. He is also Assistant Director for Research & Innovation for WAST.

Keith Couper is an associate editor of Resuscitation Plus and chair of the NIHR Research for Patient Benefit funding committee for the West Midlands region.

Harry Howe is an employee of SkyBound Rescuer.
