# Supplementary material for: The use of drone-delivered Automated External Defibrillators in the emergency response for out-of-hospital cardiac arrest. A simulation study
Source: Resusc Plus. 2025 Jul 25;25:101045. doi: 10.1016/j.resplu.2025.101045 (PMC12355114; doi:10.1016/j.resplu.2025.101045)
Supplement: Supplementary Data 1 [file mmc1.docx]

**Electronic Supplementary Material**

1. **Simulation Day Study Timings**
2. **Questionnaire (based on System Usability Scale)**
3. **Questionnaire (based on System Usability Scale) – individual participant scores**
4. **Interview topic guide**
5. **Field notes**
6. **Themes**
7. **Simulation Day Study Timings**

| Participant number | #1 | #2 | #3 | #4 | #5 | #6 | #7 | #8 | #9 | #10 | #11 |
| --- | --- | --- | --- | --- | --- | --- | --- | --- | --- | --- | --- |
| Age, male/female | 26M | 62F | 79M | 51M | 54F | 72F | 82M | 26M | 21M | 66M | 68M |
| Flight distance (km) | 1.282 | 1.276 | 1.165 | 1.159 | 1.172 | 1.157 | 1.177 | 1.167 | 1.166 | 1.171 | 1.235 |
| Emergency call to incident allocation (mm:ss) | 01:59 | 02:35 | 01:38 | 01:28 | 01:14 | 02:19 | 01:23 | 01:28 | 01:28 | 01:23 | 01:24 |
| Emergency call to take-off (mm:ss) | 02:49 | 03:25 | 02:26 | 02:18 | 02:02 | 03:08 | 02:11 | 02:18 | 02:16 | 02:11 | 02:16 |
| Flight-time (mm:ss) | 02:21 | 02:22 | 02:17 | 02:15 | 02:10 | 02:11 | 02:19 | 02:14 | 02:28 | 02:27 | 02:27 |
| Total time emergency call until arrival on scene (mm:ss) | 05:10 | 05:47 | 04:43 | 04:33 | 04:12 | 05:19 | 04:30 | 04:32 | 04:44 | 04:38 | 04:43 |
| Arrival on scene until 'safe to approach' (mm:ss) | 01:47 | 02:46 | 02:33 | 01:46 | 01:40 | 01:51 | No delivery | 01:48 | 01:46 | 01:08 | No delivery |
| Time away from patient (mm:ss) | 00:10 | 00:21 | 00:21 | 00:19 | 00:12 | 00:33 | No delivery | 00:13 | 00:13 | 00:16 | No delivery |
| Hands-off CPR time (mm:ss) | 02:41 | 02:03 | 02:58 | 01:21 | 03:13 | 02:46 | No delivery | 02:32 | 01:33 | 02:01 | No delivery |
| Arrival on scene to AED attached (mm:ss) | No AED attached | 04:43 | 05:18 | 02:22 | No AED attached | 04:26 | No delivery | 03:46 | 02:20 | 04:10 | No delivery |
| Arrival on scene to first shock (mm:ss) | No AED attached | 04:56 | 05:37 | 03:12 | No AED attached | 04:36 | No delivery | 04:06 | 02:37 | 04:35 | No delivery |
| Total time emergency call to first shock (mm:ss) | No AED attached | 10:43 | 10:20 | 07:45 | No AED attached | 09:55 | No delivery | 08:38 | 07:21 | 09:13 | No delivery |

1. **Questionnaire (based on System Usability Scale)**

**Post-event questionnaire** PARTICIPANT NUMBER________

You have just retrieved a defibrillator delivered by a drone. Regarding this (please tick the relevant box):

- 1. **I found it unnecessarily complex**

Strongly Strongly

disagree agree

|  |  |  |  |  |
| --- | --- | --- | --- | --- |
| 1 | 2 | 3 | 4 | 5 |

- 1. **I thought it was easy to do**

Strongly Strongly

disagree agree

|  |  |  |  |  |
| --- | --- | --- | --- | --- |
| 1 | 2 | 3 | 4 | 5 |

- 1. **I felt very confident doing this**

Strongly Strongly

disagree agree

|  |  |  |  |  |
| --- | --- | --- | --- | --- |
| 1 | 2 | 3 | 4 | 5 |

- 1. **I would imagine that most people would be able to do this**

Strongly Strongly

disagree agree

|  |  |  |  |  |
| --- | --- | --- | --- | --- |
| 1 | 2 | 3 | 4 | 5 |

1. **Questionnaire (based on SUS System Usability Scale) – individual participant scores**

| Participant | | I found it unnecessarily complex | I thought it was easy to do | I felt very confident doing this | I would imagine that most people would be able to do this |
| --- | --- | --- | --- | --- | --- |
| 1 | 26M | 1 | 4 | 3 | 2 |
| 2 | 62F | 1 | 4 | 5 | 5 |
| 3 | 79M | 1 | 5 | 5 | 3 |
| 4 | 51M | 1 | 2 | 1 | 4 |
| 5 | 54F | 2 | 3 | 1 | 2 |
| 6 | 72F | 1 | 2 | 2 | 3 |
| 7 | 82M | 1 | 4 | 5 | 5 |
| 8 | 26M | NA | NA | NA | NA |
| 9 | 21M | 1 | 5 | 4 | 4 |
| 10 | 66M | 2 | 3 | 3 | 3 |
| 11 | 68M | NA | NA | NA | NA |

1. **Interview Topic Guide**

**Post-event conversation questions**

PARTICIPANT NUMBER­­­________

Age (in years) _________

Gender (M, F, identifies another way – specify) ­­­_________

Training in last five years (Yes/No)

CPR only _________

AED only _________

Both _________

Real-world experience in last five years (Yes/No, number of occasions if yes)

CPR only _________

AED only _________

Both _________

*Opening question:*

- “What are your thoughts on how that went?”

*Probing questions (if needed):*

- “Did you encounter any difficulties in retrieving the defibrillator?”
- “Is there something that would make it easier for you to retrieve the defibrillator”

*Closing question:*

- “Is there something else you would like to add to your answers so far?”

1. **Field notes**

Note I scribbled these notes and transcribed here same day – so there are short cuts in my notes that I have expanded on here. They are contemporaneous enough that I am satisfied the below is accurate.

- A fair bit of drone noise after delivery even when it has ascended and before it returns to base
- Could explore giving instruction before the drone arrives (e.g. at a set distance away) to prepare the rescuer before noise makes hearing further instruction difficult
- ‘Safe to approach’ was announced when the winch had been fully retracted – is there scope to announce this earlier (e.g. at a set height above the ground)
- Noted that there is video on the drone – would participant knowing they could be seen (by call-handler?) be of use to them psychologically?
- Noise of drone – could explore giving instruction before the drone arrives (e.g. at a set distance away) to prepare the rescuer before noise makes hearing further instruction difficult
- Welsh Ambulance Service NHS Trust (indeed, all UK ambulance services) do not device-specific instructions for AED (as they are all different) and so couldn’t give instructions on our device.
- There was the added complication of having to ‘slide out’ the AED from the bespoke AED carrier that, to our experience, is an unusual way to open the device
- There were no hesitations about leaving the patient. Note that this was an outdoor location, could hear the drone on its approach and – if participants orientated the right way – could see it on its approach and as it arrived.
- There was no ‘abandon mission’ communication set-up between drone-handler and call-handler in this simulation, so could not tell the participant that the drone was not going to arrive after all (we have back-up comms to do this, and ended scenarios when this happened).

**CALL-HANDLER FEEDBACK**

- Opening defib casing - some volunteers struggled to open the case of the defib. Instructions could be included in the script so that they are able to open the defib quickly and effectively.
- Drone failure - In case of drone failure/recall, there should be an alternative script included just so that the caller(s) is still in the loop with what's happening with the defib and whether they will still be receiving one.
- Volunteers - I thought two of the volunteers were testing me rather than just the scripting for the defib due to them cutting me off or speaking over me. I can understand that whilst this may happen with members of the public, it wasn't necessary for this training. [Ed – this was NOT the case]
- The “Wait” time between landing the Defib drone and the safe to approach time is a consistently around 2 minutes. These can obviously be vital in a real life and death situation and also, could cause confusion and distress in a real-world situation. If the rescuer is a loved one of the patient, they will see the DefibDrone land and are then told they can’t get it yet, then having to wait while looking at the device that, in their potentially emotional and irrational mindset, is the thing that will save their loved ones life.
- I understand the need to ensure the drone is safe to approach as to avoid injuries (we don’t need another patient) but could this be looked into, for a potential reduction in this wait time, to not only improve the response to the patient but also reduce the stress on the rescuer or the potential that the rescuer will ignore the instruction to wait and try and collect the defib before its safe.

**PARTICIPANTS**

Flight 1

*Participant*

- Scenario was realistic – I forgot I was doing a simulation
- Putting phone on speaker would have helped me. It was not my own phone so I could not work out how to do it
- It was difficult to hear instructions subsequently
- CPR instructions (from the call-handler) were excellent
- Defibrillator – its arrival worked well. It was place well and there were no difficulties in getting it
- I couldn’t open it initially – looked like box with a lid; I could see how people would get flustered in real life
- Defibrillator pads – it was unclear if the pads were universal / interchangeable
- Defibrillator instructions – it didn't say straight on [Ed – bare] chest initially; I put it on the  clothes, and the call-handler helped clarify need for putting on bare skin
- The drone did not distract me from doing CPR
- Some more background detail in the simulation pre-brief would have helped – e.g. where we were / address

*Researcher review (Researcher review and noted on scene)*

- The participant held the phone to their ear whilst getting instruction
- Participant listened to the entire instruction before putting the phone down – they nearly put it down at one point but the instruction continued so they kept it at their ear. They put ONE of their hands on the manikin’s chest whilst holding phone still
- Counted out “1,2,3,4” – had phone on floor so was bent right down to speak into phone (“1,2,3,4”) whilst doing CPR
- Said “OK” when told the defibrillator was coming over phone, but asked “do you want me to continue CPR?”
- They occasionally looked up on first hearing drone noise, occasionally looked up thereafter
- Said “I can hear the drone coming in”
- Said “OK” when told not to leave patient and then, “so, am I continuing CPR?”
- After this, kept phone in ear, so no longer bending face to ground
- Said “Defib is on the ground, awaiting your instructions”, before told it was safe to approach
- There was no delay when they were told to get defib, took phone with them, talked through (I’m leaving, I have defib etc.) all the way through this journey. Ran there and back.
- AED:
- “Are instructions on the defibrillator”
- Did a few compressions whilst trying to get defibrillator on.
- They placed the AED pads on top of jacket first, responded to “on bare chest” instructions, said “bare chest” to self out loud and corrected
- Mostly listening to AED instructions, little input here from call-handler
- The participant was facing the direction that the drone came from – they told call-handler that “the drone is on the way” and spent a lot of time looking at it as it lowered (whilst doing CPR)
- Winch lowering at same time as drone lowered – seemed quicker?
- Did not give shock (did not get pads off sticky backs). Discussed with call handler and came to agreement to re-start CPR. Restarted, scenario ended.

Flight 2

*Participant*

- Excellent. I would be so relieved if I was in the middle of nowhere. It’s logical in that situation too – why do CPR if there is no AED available?
- The AED was intuitive. Pictures and instructions were useful [Ed – AED has a digital display showing actions e.g. putting on pads]. Pictures most helpful
- Knees wobbly when got up after a while to get AED. Scared to run – accept that walking and costing a few seconds would make little difference to patient but running and falling might
- It would be easier if AED was closer
- Drone – I was worried it might land ON me. Initially thought that might fly OVER me so I could see it. Being told, drone is on its way, it will land NEAR you and not fly OVER you might have helped

*Researcher review*

- They were facing away from drone approach
- Some back and forth re: address, took a long time to confirm location. They spelled out [Ed – the long] name of nearest village
- Phone was to their ear whilst getting instruction, finished instruction before putting phone down. They put their first hand on chest whilst still holding the phone
- They gave clear ‘no’ answers to breathing / conscious questions. Said “looks like a cardiac arrest”
- Said “OK” to ‘drone was on its way’ and ‘don’t leave patient’ statements, no clarification needed
- They briefly look up when they initially heard the drone noise.
- They looked at the AED once when it was on ground
- They left patient quickly when told, didn’t confirm that it was okay to do so with the call-handler, didn’t take phone with them.
- Walked, didn’t run, confirmed when they were back with the patient
- AED:
- Said “Where’s the on button” and then quickly found it
- Looked briefly at AED before coming back to patient
- Didn’t try to open AED until getting instruction to open by call-handler
- Found no problems opening the AED in the end

Flight 3

*Participant*

- Could anybody do this? Not sure they could if they were older, not as fit – they couldn't do CPR for as long as in the scenario. Perhaps they’d get the energy from somewhere
- AED – it was not obvious how to open using the mechanism. I didn’t open yellow tab at first. There was no specific instruction from call-handler. A specific instruction would help: e.g. “open tab” “lift out”
- I followed defibrillator instructions ahead of those given by ambulance service
- There was no problem with drone noise; I’m “used to more noise” [Ed – because of previous professional background, disclosed pre-simulation]

*Researcher review*

- Participant was facing away from direction of drone approach
- They started CPR briefly, but stopped as soon as given phone and didn’t resume, even after telling call-handler “no pulse”
- “Defib on way to you” – said “OK”, but stopped CPR briefly to listen and answer
- Started off with “1,2,3,4” but stopped saying this out loud after first interruption from call-handler (who said the defibrillator was on the way)
- They briefly looked up and around when drone first arrived.
- Said “Drone has arrived” – “can’t hear you”, stopped CPR to pick up phone to confirm, then “OK”
- They had to pick up phone to hear “go to get AED” instruction (Drone noise), stopped CPR to do it. “OK” and then left to get AED without taking phone
- Slowly jogged towards the AED
- AED:
- Initially started trying to open AED, then picked up phone again.
- They then only tried to open it once they had received all instructions about the AED from the call-handler
- Had to put phone down, needed both hands, took a while to work out how to open box
- Once AED started talking – said “carry on” to the call-handler.
- Once pads on, picked up phone, listened to instructions, said “yes, have done this” as they (and the AED) were a few steps ahead of the call-handler
- Pads were on, call-handler gave instructions about putting pads on so touched them again just before AED analyses
- Conflict between device / call-handler, as above

Flight 4

*Participant*

- The drone was loud. I couldn’t hear call-handler, even when on speaker
- It was frustrating seeing the defibrillator hanging on the winch, not being able to go and get it
- Once I got the device, I worked it out okay but wondered if others would be able to work it out
- AED – good when long response time, “nice”; I know in this area that “a truck [Ed – ambulance] could be 20 mins away”
- AED – it was obvious what it was. “Not complicated”
- Drone – was surprised to see AED on winch. I had assumed it would land and I’d retrieve AED from the top of the drone
- Even if I had access to an AED nearby I would be tempted to stay with patient / do CPR and wait for the drone-delivered version
- I know not to stop CPR but the AED being winched down was a tease – would someone else be tempted just to go and grab it?

*Researcher review*

- They started CPR spontaneously, doing CPR whilst on the phone, with phone by their side
- They specifically said that they were alone and they had “no gear” with them, was doing “active CPR”
- Facing direction of drone approach
- Said “OK” and “OK thank you” when told drone was on the way, and did not seek clarification
- They asked for a metronome tone from call-handler
- Said “I can see a drone in the distance” – answered “OK” by call-handler.
- Said “Drone is flying towards me”, and said out loud that it was coming “to right location”
- Said “I understand” to the ‘don’t leave the patient’ prompt.
- Periodically looked up at drone and AED being lowered, continued CPR
- Said “Are you still there” a few times to the call-handler, in response to periods of silence
- AED:
- Ran towards the AED, walked back whilst working out how to open it
- Opened it without prompt
- Didn’t announce their return to the call-handler
- Said “Pads are on, just waiting for AED to sort itself out”
- Said “Analysing” to call-handler
- So, no need for call-handler intervention
- In the hands a very competent individual, call-handler did very well in supporting role, not intervening unnecessarily.
- The participant had experience and was well-practised in CPR/AED
- They scraped their finger on ground whilst retrieving AED from ground
- There was a lot of quiet/down time with call handler. The participant twice asked “are you still there” - the second time was when the drone winch was retracting after AED had landed

Flight 5

*Participant*

- It would be quite a panic in a real-life situation
- It was difficult to hear call-handler because of the noise of the drone – I do not think the phone was on speaker [Ed – it was]
- I lost “vital” time trying to open AED. The call handler gave instructions but I had “no idea”. They need to assume zero knowledge and give “noddy” instructions
- I could see drone coming
- I was worried about leaving the patient – I was told to continue compressions and how could I do that whilst leaving the patient. So, I ran as fast as I could
- There was a lot of back and forth with the call-handler about instructions for chest compressions. This was partly because I was trying to concentrate on skill and partly because the instructions were not clear
- You need to be told things exactly, e.g.
- The defibrillator is in a box that will land NEAR you
- It will be in a red box (use of colours over phone would be helpful)
- You need to leave patient and get it
- I can see how AED would work, but I can’t see how *I* could make it work [Ed – with how they performed / struggled to operate it]
- I had no problem with interacting with the drone, even though I had never seen or heard one before.
- The drone “whir” took me by surprise – I couldn't see how I could hear phone without holding it to my ear, but then how could I do effective chest compressions?

*Researcher review*

- They were facing in the direction of the drone’s approach throughout
- They held the phone to ear until starting to give CPR
- Pressured speech throughout, unclear if this was real or trying to ‘act’ for the scenario.
- They often spoke over the call-handler
- They only started CPR once instructions had being given; then asked for CPR instructions to be repeated as they were actually doing it
- Said: “Is there a speaker”, didn’t wait for answer, then “can you speak really loudly” please
- Shouting at several points
- Asked the call-handler, “I press every time I count?” (“1,2,3,4”) several times
- Said “I’m not sure I’m doing this right”
- Said, “Drone? Christ” to drone is coming instruction and “is an ambulance coming”
- Said “I need you to talk louder” when asked not to leave patient.
- They kept looking at the drone during its approach
- Said “How am I going to get over there” when seeing AED lowering
- Said “I’m taking you with me” “I have to leave the patient, right” – when given instruction to do so by call-handler, but still left reasonably quickly,
- AED:
- Said “What the hell do I do with this” “I’ve never seen one of these before
- Said “Can you shout that” (when call-handler was giving AED instructions)
- Said “How do I open it”, several times. Call-handler unclear how to give specific instructions
- Got it open after much delay
- Said “It says plug and apply electrode. I’ve got a box, some pads and some wires”
- They really struggled to open the AED box
- They asked clarification questions A LOT to the call-handler
- There were AED issues – the device advanced, advised shock before pads attached. This confused the participant. The shock was cancelled after delay...the scenario ended with some confusion

Flight 6

*Participant*

- I couldn’t hear [Ed – throughout, not just a drone noise issue] – I felt would have put own phone on speakerphone [Ed – our phone WAS on speakerphone].
- I had to put the phone near my ear, which was difficult to do whilst performing CPR
- Imagining this happening is scary
- It was simple enough to do
- It felt good when I could see the drone – gave me confidence that the AED was on its way
- I had difficulty opening AED – it would have been nice to know it was a sliding door
- The call-handler said “open” the AED; would be better to have the words “slide” or “pull here” on it in the relevant areas
- I pulled out yellow tab – put it back in again as not sure if I should have or not
- There is nothing in particular to make things easier, just slightly difficult to get up off knees to get the defibrillator

*Researcher review*

- Was “going to do two sessions” and then “two minutes CPR” first, pretending to give rescue breaths – we actively intervened to give phone as there was no apparent endpoint otherwise
- They were facing direction of drone approach
- They stopped doing their spontaneously initiated CPR as soon as they got the phone, and kept phone to their ear throughout, and did not restart until the completion of CPR instructions
- Before the completion of the CPR instructions, they were trying to put their hand on chest and keep phone in ear
- They then put phone down on patient rather than the ground
- “Doing really well” “Are you ok” a few times from call-handler, unprompted, and “keep going”
- Said “That’s brilliant, thank you”, when told drone on its way, with no clarification questions asked
- In response to the call-handler saying, “Shouldn’t be too long” the participant said “I can see it now”
- Said “There it is”, was told not to leave patient, said “no, okay”, and no further clarification sought by participant. Said: “Its nearly on the ground” [Ed – true]
- They Looked up a lot as AED was being lowered down, but didn’t leave patient until told
- They walked to the AED, took phone with them, kept it to their ear
- Said: “I’m back to the patient”
- AED:
- Holding phone to ear whilst dealing with AED
- “How does it open” x 2 but call-handler “don’t have specific instructions
- Took a little while, but worked it out
- Putting pads on just ahead of being given instructions
- “Machine will tell you what to do” but patient had already pressed shock button and machine was telling them to re-start CPR
- We ended the scenario as soon as the participant re-started CPR after delivering a shock, but the call-handler was still quite behind with AED-related instructions, so didn’t get chance to see if call-handler would follow-up with further AED instructions or if they’d realise that CPR had re-started.

Flight 7

*Participant*

- I couldn’t hear the call-handler well. [Ed – our phone was on speakerphone]. If I had my own phone, I believe I could have manipulated the phone to help
- It certainly seemed that the AED arrived swift – faster than ambulance in any case
- I couldn’t get up from knees after ending scenario – if had been life-and-death I would have crawled
- The drone position seemed very accurate, it seemed as if it was right overhead
- The drone – you are reliant on this in a rural location, you are not able to get someone else to you there to help you.

*Researcher review*

- The participant was facing direction of drone approach
- They were holding phone to ear a lot
- Kept phone to ear from throughout getting CPR instruction
- Put first hand on chest during instruction, whilst holding phone to ear
- Then put down and started CPR – didn’t count out loud or acknowledge that they had started CPR
- They had difficulty hearing call-handler, so every time that the call-handler spoke the participant had to stop and put the phone to their ear
- There was some technical issue so the call-handler not aware if drone was coming initially: the participant picked up on this, picked up phone to tell the call-handler drone was on site
- They would stop CPR / doing CPR one-handed for a period of time, when trying to listen to call-handler speech
- The drone was not able to land (emergency ‘Return to Base activated’ automatically). Given patient had to be helped from knees we decided NOT to re-run the scenario [Ed – could not justify this on ethical/safety grounds]. We did not get to the point of trying to open the AED
- It was clear before the scenario started that the participant had a degree of hearing impairment to begin with

Flight 8

*Participant*

- The operator did not say to keep doing CPR
- When I brought the package [Ed – AED] back from drone and was back at the patient’s side I asked “Do I need to continue CPR”
- When I initially rung 999 – there was a delay to mentioning CPR
- I had no difficulties getting the defibrillator
- It was clear about how to open box – it was written on the box to pull yellow tab [Ed – actually, it wasn’t]
- I misheard call-handler instructions because of the defibrillator instructions
- Generally, it worked well

*Researcher review*

- They were facing away from direction of drone approach
- Once the participant was told to make sure the casualty was on their back (during CPR instructions), they took the phone away from ear, looking to find speaker button, put it on ground, and then started CPR only once instruction most of the way finished
- Said “OK” to ‘drone dispatched to your location’, no clarification sought
- Call-handler said: “I’ll let you know when defib is on scene, keep going”
- Said “Yep”, in response to drone on scene / don’t leave patient, no clarification sought
- The drone ended up slightly to their side, so they able to look over shoulder at landing point
- They ran to the defibrillator, took the phone with them, held by side
- Said: “I’ve got the defibrillator”
- Call-handler asked “Is there someone there to help you?”
- They waited for call-handler to start AED instructions. There was a slight delay from the call-handler here, “bear with me two seconds”
- AED:
- They didn’t try to open box themself, waited for the instructions
- They Got ahead of call-hander
- The call-handler said: “The machine will tell you what to do, follow its instructions” – participant then had to say that the AED had advised shock and that the shock had been delivered.
- Call-handler understood, and moved quickly to restart CPR instructions, specific instruction to follow metronome beat on AED. There was a delay to restarting CPR
- AED use was incorrect: plugged in the connector before attaching pads, so it decided to shock before pads in position

Flight 9

*Participant*

- It was good, simple, straightforward; as long as weather conditions allow. I managed this despite the wind / drizzle. I think consumables might blow away in wind if doing outdoors in real life
- Speed saves lives
- It would be great if the drone landed closer, but I appreciate the need for safety
- Would it work as well in inaccessible / difficult terrain where couldn’t land AED as close (e.g. mountainous area)?
- I heard the drone from a while away. This is potentially distracting but the noise was only for a short period of time, so not a big issue
- Knowing that the drone was coming was reassuring – hearing the “whirring” of the drone is a bit like hearing the ambulance sirens and knowing help is on the way
- Does it need a blue light or siren?

*Researcher review*

- They were facing towards direction of drone approach
- They were holding phone in front of them, rather than at ear, but still holding in one hand
- As soon as they were told to place hands on chest, they put phone down, but still didn’t start CPR instruction until the instruction had been completed
- Said: “OK” and “gotcha” in response to ‘drone on its way’ and ‘don’t leave the patient’ instructions
- They looked up at drone at lot, facing AED directly once it landed and watched it for a while
- They left the patient as soon as they were told to, took phone with them, ran to defibrillator
- AED:
- Said “I’ve got it”
- Opened quickly once following instructions
- Said: “Chest is bare” “machine is on” in response to prompts from call-handler
- The call-handler said “Follow instructions” after the AED had told participant to deliver a shock, so they just replied “pressed the button”. “Is shock delivered” asked the call-handler; participant said “yes” and there was immediate instruction from the call-handler to carry on CPR
- The sticky back from AED pads blew away as they opened it. They tried to grab it, initially but did not chase it

Flight 10

*Participant*

- I found the simulation confusing, I was unsure how much to treat as if a real-life situation
- I could hear drone arriving
- I didn’t look up – but I could see the reflection of it hovering in phone; I chose to ignore
- I had no problems getting the defibrillator
- The defibrillator could have landed closer by
- I was initially at a loss about what to do with the defibrillator – took a while to work out that had to pull the yellow lever to open the box. It was not at all obvious
- Interacting with the call-handler was distracting whilst trying to concentrate on CPR.
- I had no issues with sound during the phone call

*Researcher review*

- Note that they started CPR and showed no sign of making a 999 call, so we intervened and provided the phone
- They were facing toward the direction of drone approach
- When the 999 call was answered, the participant said ‘hello’, put the phone down and continued with CPR, with rescue breaths (doing around 10-12:1 chest compression to ventilation ratio
- They occasionally stopped when concentrating on call-handler voice
- Said “yes” when offered CPR instruction, but carried on regardless with CPR and rescue breaths. They didn’t specifically say to the call-handler that they were doing CPR
- Said “okay” when told the drone on the way
- There was “Well done, keep going” from call-handler at one point, to which the participant replied “yeah, okay”
- The drone arrived, hovered well above usual height, and so there was a very long time until got the “arrived on scene / stay with patient / don’t approach” instructions. We did not have the means to communicate to the call-handler that there was a delay here.
- The participant didn’t look up at the drone or at where the AED had landed at any time
- AED:
- They retrieved it without difficulty
- Pads attached, but through clothes, and they didn’t respond / fix after “bare chest” prompt from the defibrillator, so, recorded as shock delivery, but inadequate / poor shock delivery

Flight 11

*Participant*

- I could hear the drone moving around above me
- I could tell there was an issue based on motor noise varying
- I didn’t look up – I was concentrating on the patient
- I heard the drone leave – assumed it had landed initially and was waiting to hear about how to get AED (I hadn’t seen it land) [Ed – in fact the drone had initiated an emergency / automated Return to Base]
- I could see AED when it was being winched down [Ed – the winching/lowering process had started before mission abort] and was waiting for it to land

*Researcher review*

- They were facing the direction of drone approach
- They were holding the phone to their ear whilst getting CPR instruction
- They let the call-handler finish giving CPR instruction before putting phone down
- Said “OK” to “defib coming”, no clarification sought
- Said: “That’s fine” when told not to leave patient, no clarification sought
- They didn’t look up at any time on drone arrival
- They looked up briefly when the AED on end of winch, when it was approximately head height

This simulation was abandoned. Initial problems with winch lowering and then there was interference / possible nearby airspace ingress so the drone triggered an emergency Return to Base procedure

1. **Themes**

*General researcher observations – notes made during the simulation days, not related to a specific simulation*

*#, participant = observations made by that participant in post-event interview*

*#, researcher – observations made by researcher on viewing the simulation, and after reviewing the 999-call audio and simulation video afterwards*

1. Communication between call-handler and bystander
   1. The effect of drone noise on communications

General researcher observations: A fair bit of drone noise after delivery even when it has ascended and before it returns to base

General researcher observations: could explore giving instruction before the drone arrives (e.g. at a set distance away) to prepare the rescuer before noise makes hearing further instruction difficult

#3, participant: There was no problem with drone noise; I’m “used to more noise” [Ed – because of previous professional background, disclosed pre-simulation]

#3, researcher: They had to pick up phone to hear “go to get AED” instruction (Drone noise), stopped CPR to do it. “OK” and then left to get AED without taking phone

#4, participant: The drone was loud. I couldn’t hear call-handler, even when on speaker

#5, participant: It was difficult to hear call-handler because of the noise of the drone – I do not think the phone was on speaker [Ed - it was]

#5, participant: The drone “whir” took me by surprise – I couldn't see how I could hear phone without holding it to my ear, but then how could I do effective chest compressions?

- 1. Discussions about retrieving the AED

#1, researcher: Said “OK” when told the defibrillator was coming over phone, but asked “do you want me to continue CPR?”  Said “I can hear the drone coming in”. Said “OK” when told not to leave patient and then, “so, am I continuing CPR?” Said “Defib is on the ground, awaiting your instructions”, before told it was safe to approach

#4, researcher: Said “OK” and “OK thank you” when told drone was on the way, and did not seek clarification. Said “I can see a drone in the distance” – answered “OK” by call-handler. Said “Drone is flying towards me”, and said out loud that it was coming “to right location”. Said “I understand” to the ‘don’t leave the patient’ prompt

# 5 participant: You need to be told things exactly, e.g. The defibrillator is in a box that will land NEAR you. It will be in a red box (use of colours over phone would be helpful). You need to leave patient and get it

#6, researcher: Said “There it is” [Ed – about the drone, as it was approaching], was told not to leave patient, said “no, okay”, and no further clarification sought by participant.

- 1. Liaising with call-handler to retrieve and use the AED

#1, researcher: Mostly listening to AED instructions, little input here from call-handler

#3, participant: I followed defibrillator instructions ahead of those given by ambulance service

#3, researcher: Initially started trying to open AED, then picked up phone again. They then only tried to open it once they had received all instructions about the AED from the call-handler

#3, researcher: Once AED started talking – said “carry on” to the call-handler. Once pads on, picked up phone, listened to instructions, said “yes, have done this” as they (and the AED) were a few steps ahead of the call-handler*.* Pads were on, call-handler gave instructions about putting pads on so touched them again just before AED analyses

#4, researcher: Opened AED without prompt. Said “Pads are on, just waiting for AED to sort itself out.” Said “Analysing” to call-handler. So, no need for call-handler intervention - in the hands a very competent individual, call-handler did very well in supporting role, not intervening unnecessarily.

#5, researcher: AED: Said “What the hell do I do with this” “I’ve never seen one of these before. Said “Can you shout that” (when call-handler was giving AED instructions. Said “How do I open it”, several times. Call-handler unclear how to give specific instructions. Got AED open after much delay. Said “It says plug and apply electrode. I’ve got a box, some pads and some wires”

#6, researcher: We ended the scenario as soon as the participant re-started CPR after delivering a shock, but the call-handler was still quite behind with AED-related instructions

#6, researcher: Participant was putting pads on just ahead of being given instructions*. The call handler said* “Machine will tell you what to do” but patient had already pressed shock button and AED was telling them to re-start CPR

#8, researcher: Said: “I’ve got the defibrillator.” Call-handler asked “Is there someone there to help you?” They waited for call-handler to start AED instructions before trying to open box. There was a slight delay from the call-handler here, “bear with me two seconds”

#8, participant: I misheard call-handler instructions because of the defibrillator instructions

#8, researcher: AED: They didn’t try to open box themself, waited for the instructions*.* They got ahead of call-hander*.* The call-handler said: “The machine will tell you what to do, follow its instructions” – participant then had to say that the AED had advised shock and that the shock had been delivered. Call-handler understood, and moved to restart CPR instructions, specific instruction to follow metronome beat on AED. There was a delay to restarting CPR.

#9, researcher: AED: Said “I’ve got it” Opened quickly once following instructions Said: “Chest is bare” “machine is on” in response to prompts from call-handler. The call-handler said “Follow instructions” after the AED had told participant to deliver a shock, so they just replied “pressed the button”. “Is shock delivered” asked the call-handler; participant said “yes” and there was immediate instruction from the call-handler to carry on CPR

- 1. Other communication with 999 call-handler

#1, participant: Scenario was realistic – I forgot I was doing a simulation. Putting phone on speaker would have helped me. It was not my own phone so I could not work out how to do it.

#1, participant: It was difficult to hear instructions subsequently [Ed - as the phone was not on speaker]

#1, researcher: The participant held the phone to their ear whilst getting instruction. Participant listened to the entire instruction before putting the phone down – they nearly put it down at one point but the instruction continued so they kept it at their ear. They put ONE of their hands on the manikin’s chest whilst holding phone still. Counted out “1,2,3,4” – had phone on floor so was bent right down to speak into phone (“1,2,3,4”) whilst doing CPR

#2, participant: Phone was to their ear whilst getting instruction, finished instruction before putting phone down. They put their first hand on chest whilst still holding the phone

#2, researcher: Some back and forth re: address, took a long time to confirm location. They spelled out [Ed – the long] name of nearest village, delayed onset of CPR instructions

#2, researcher: They gave clear ‘no’ answers to breathing / conscious questions. Said “looks like a cardiac arrest”. Said “OK” to ‘drone was on its way’ and ‘don’t leave patient’ statements, no clarification needed

#3, researcher: Started CPR briefly, but stopped as soon as given phone and didn’t resume, even after telling call-handler “no pulse”

#3, researcher: “Defib on way to you” – said “OK”, but stopped CPR briefly to listen and answer

#3, researcher: Started off counting “1,2,3,4” but stopped saying this out loud after first interruption from call-handler (who said the defibrillator was on the way)

#3, researcher: They had to pick up phone to hear “go to get AED” instruction (Drone noise), stopped CPR to do it. “OK” and then left to get AED without taking phone

#3, researcher: started CPR spontaneously but then stopped when talking to call-handler and did not start again until specific CPR instructions were given

#4, researcher: They started CPR spontaneously, doing CPR whilst on the phone, with phone by their side

#4, researcher: started CPR spontaneously and carried on when 999 call answered.

#5, researcher: They held the phone to ear until starting to give CPR

#5, participant: There was a lot of back and forth with the call-handler about instructions for chest compressions. This was partly because I was trying to concentrate on skill and partly because the instructions were not clear

#6, participant: I couldn’t hear [Ed – throughout, not just a drone noise issue] – I felt would have put own phone on speakerphone [Ed – our phone WAS on speakerphone]. I had to put the phone near my ear, which was difficult to do whilst performing CPR

#6, participant: I couldn’t hear [Ed – throughout, not just a drone noise issue] – I felt would have put own phone on speakerphone [Ed – our phone WAS on speakerphone]. I had to put the phone near my ear, which was difficult to do whilst performing CPR

#6, researcher: started CPR spontaneously but then stopped when talking to call-handler and did not start again until specific CPR instructions were given

#6, researcher: They stopped doing their spontaneously initiated CPR as soon as they got the phone, and kept phone to their ear throughout, and did not restart until the completion of CPR instructions*.* Before the completion of the CPR instructions, they were trying to put their hand on chest and keep phone in ear. They then put the phone down on patient rather than the ground.

#7, researcher: They were holding phone to their ear a lot: Kept phone to ear from throughout getting CPR instruction; Put first hand on chest during instruction, whilst holding phone to ear; Then put down and started CPR – didn’t count out loud or acknowledge that they had started CPR

#7, researcher: They had difficulty hearing call-handler, so every time that the call-handler spoke the participant had to stop and put the phone to their ear

#7, researcher: They would stop CPR / doing CPR one-handed for a period of time, when trying to listen to call-handler speech

#7, participant: I couldn’t hear the call-handler well. [Ed - our phone was on speakerphone].

#7, researcher: They had difficulty hearing call-handler, so every time that the call-handler spoke the participant had to stop and put the phone to their ear

#7, researcher: They would stop CPR / doing CPR one-handed for a period of time, when trying to listen to call-handler speech

#8, researcher: Once the participant was told to make sure the casualty was on their back (during CPR instructions), they took the phone away from ear, looking to find speaker button, put it on ground, and then started CPR only once instruction most of the way finished

#8, researcher: Said “OK” to ‘drone dispatched to your location’, no clarification sought*.* Call-handler said: “I’ll let you know when defib is on scene, keep going”*.* Said “Yep”, in response to drone on scene / don’t leave patient, no clarification sought

#9, researcher: They were holding phone in front of them, rather than at ear, but still holding in one hand. As soon as they were told to place hands on chest, they put phone down, but still didn’t start CPR instruction until the instruction had completed

#9, researcher: Said: “OK” and “gotcha” in response to ‘drone on its way’ and ‘don’t leave the patient’ instructions

#10, participant: Interacting with the call-handler was distracting whilst trying to concentrate on CPR. I had no issues with sound during the phone call

#10, researcher: started CPR spontaneously and carried on when 999 call answered.

#10, researcher: When the 999 call was answered, the participant said ‘hello’, put the phone down and continued with CPR, with rescue breaths (doing around 10-12:1 chest compression to ventilation ratio

#10, participant: Interacting with the call-handler was distracting whilst trying to concentrate on CPR. I had no issues with sound during the phone call

#10, participant: Interacting with the call-handler was distracting whilst trying to concentrate on CPR.

#10, researcher: They occasionally stopped when concentrating on call-handler voice. Said “yes” when offered CPR instruction, but carried on regardless with CPR and rescue breaths. They didn’t specifically say to the call-handler that they were doing CPR. Said “okay” when told the drone on the way. There was “Well done, keep going” from call-handler at one point, to which the participant replied “yeah, okay”

#11, researcher: They were holding the phone to their ear whilst getting CPR .  let the call-handler finish giving CPR instruction before putting phone down

#11, researcher: Said “OK” to “defib coming”, no clarification sought. Said: “That’s fine” when told not to leave patient, no clarification sought

1. Communication between call-handler and drone operator

General researcher observations: there was no ‘abandon mission’ communication set-up between drone-handler and call-handler, so could not tell the participant that the drone was not going to arrive after all (we have back-up comms to do this, and ended scenarios when this happened)

Call-handler feedback: Drone failure - In case of drone failure/recall, there should be an alternative script included just so that the caller(s) is still in the loop with what's happening with the defib and whether they will still be receiving one.

#10, researcher: The drone arrived, hovered well above usual height, and so there was a very long time until got the “arrived on scene / stay with patient / don’t approach” instructions. We did not have the means to communicate to the call-handler that there was a delay here.

1. Interaction with the drone
   1. Drone as a distraction

General researcher observations: Facing away from drone as it approached: #1, #2, #3, #8, #11; facing towards drone as it approached: #4, #5, #6, #7, #9, #10

#1, participant: The drone did not distract me from doing CPR

#1, researcher: They occasionally looked up on first hearing drone noise, occasionally looked up thereafter. The participant was facing the direction that the drone came from – they told call-handler that “the drone is on the way” and spent a lot of time looking at it as it lowered (whilst doing CPR)

#2, researcher: They briefly look up when they initially heard the drone noise. They looked at the AED once when it was on ground

#3, researcher: They briefly looked up and around when drone first arrived.

#3, researcher: They had to pick up phone to hear “go to get AED” instruction (Drone noise), stopped CPR to do it. “OK” and then left to get AED without taking phone

#4, researcher: Periodically looked up at drone and AED being lowered, continued CPR

#4, researcher: Said “OK” and “OK thank you” when told drone was on the way, and did not seek clarification. Said “I can see a drone in the distance” – answered “OK” by call-handler. Said “Drone is flying towards me”, and said out loud that it was coming “to right location”. Said “I understand” to the ‘don’t leave the patient’ prompt

#5, participant: The drone “whir” took me by surprise – I couldn't see how I could hear phone without holding it to my ear, but then how could I do effective chest compressions?

#5, researcher: They kept looking at the drone during its approach

#6, researcher: Said “There it is”, was told not to leave patient, said “no, okay”, and no further clarification sought by participant. Said: “Its nearly on the ground” [Ed - true]*.* They Looked up a lot as AED was being lowered down, but didn’t leave patient until told

#8, researcher: The drone ended up slightly to their side, so they were able to look over shoulder at landing point

#9, participant: I heard the drone from a while away. This is potentially distracting but the noise was only for a short period of time, so not a big issue

#9, researcher: They looked up at drone at lot, facing AED directly once it landed and watched it for a while

#10, participant: I could hear drone arriving*.* I didn’t look up – but I could see the reflection of it hovering in phone; I chose to ignore

#10, participant: The participant didn’t look up at the drone or at where the AED had landed at any time

#11, participant: I could hear the drone moving around above me. I could tell there was an issue based on motor noise varying. I didn’t look up – I was concentrating on the patient. I heard the drone leave – assumed it had landed initially and was waiting to hear about how to get AED (I hadn’t seen it land) [Ed- in fact the drone had initiated an emergency / automated Return to Base]. I could see AED when it was being winched down [Ed- the winching/lowering process had started before mission abort] and was waiting for it to land

#11, researcher: They didn’t look up at any time on drone arrival. They looked up briefly when the AED on end of winch, when it was approximately head height

- 1. Interaction with drone and retrieving AED

General researcher observations: there were generally no hesitations about leaving the patient. Note that this was an outdoor location, could hear the drone on its approach and – if participants orientated the right way – could see it on its approach and as it arrived.

#1, participant: Defibrillator – its arrival worked well. It was place well and there were no difficulties in getting it

#2, researcher: They left patient quickly when told, didn’t confirm that it was okay to do so with the call-handler, didn’t take phone with them. Walked, didn’t run, confirmed when they were back with the patient

#2, participant: It would be easier if AED was closer

#2, participant: Knees wobbly when got up after a while to get AED. Scared to run – accept that walking and costing a few seconds would make little difference to patient but running and falling might.

#3, researcher: Slowly jogged towards the AED

#4, researcher: They scraped their finger on ground whilst retrieving AED from ground

#4, participant: It was frustrating seeing the defibrillator hanging on the winch, not being able to go and get it. I know not to stop CPR but the AED being winched down was a tease – would someone else be tempted just to go and grab it?

#4, participant: Drone – was surprised to see AED on winch. I had assumed it would land and I’d retrieve AED from the top of the drone

#4, researcher: Ran towards the AED, walked back whilst working out how to open it. Didn’t announce their return to the call-handler.

#5, participant: I had no problem with interacting with the drone, even though I had never seen or heard one before.

#5, participant: Said “How am I going to get over there” when seeing AED lowering. Said “I’m taking you with me” “I have to leave the patient, right” – when given instruction to do so by call-handler, but still left reasonably quickly,

#6, researcher: They walked to the AED, took phone with them, kept it to their ear*.* Said: “I’m back to the patient”

#6, participant: There is nothing in particular to make things easier, just slightly difficult to get up off knees to get the defibrillator

#6, researcher: They had a lower limb injury – we made modifications to kneeling area / added padding to accommodate

#7, participant: I couldn’t get up from knees after ending scenario – if had been life-and-death I would have crawled

#7, researcher: It was clear before the scenario started that the participant had a degree of hearing impairment to begin with

#7, participant: The drone position seemed very accurate, it seemed as if it was right overhead

#7, researcher: There was some technical issues curtailing the scenario, meaning AED retrieval never happened. Given patient had to be helped from knees we decided NOT to re-run the scenario [Ed - could not justify this on ethical/safety grounds].

#8, participant: I had no difficulties getting the defibrillator

#9, researcher: They left the patient as soon as they were told to, took phone with them, ran to defibrillator

#9, participant: It would be great if the drone landed closer, but I appreciate the need for safety. Would it work as well in inaccessible / difficult terrain where couldn’t land AED as close (e.g. mountainous area)?

#10, participant: I had no problems getting the defibrillator.

#10, participant: The defibrillator could have landed closer by

- 1. Drone delays

General researcher observations: ‘safe to approach’ was announced when the winch had been fully retracted – is there scope to announce this earlier (e.g. at a set height above the ground)

Call-handler feedback: The “Wait” time between landing the Defib drone and the safe to approach time is a consistently around 2 minutes. These can obviously be vital in a real life and death situation and also, could cause confusion and distress in a real-world situation. If the rescuer is a loved one of the patient, they will see the DefibDrone land and are then told they can’t get it yet, then having to wait while looking at the device that, in their potentially emotional and irrational mindset, is the thing that will save their loved ones life. I understand the need to ensure the drone is safe to approach as to avoid injuries (we don’t need another patient) but could this be looked into, for a potential reduction in this wait time, to not only improve the response to the patient but also reduce the stress on the rescuer or the potential that the rescuer will ignore the instruction to wait and try and collect the defib before its safe.

1. Using the AED
   1. Accessing the AED

General researcher observations: Welsh Ambulance Service NHS Trust do not device-specific instructions for AED (as they are all different), and so couldn’t give instructions on our device.

General researcher observations: There was the added complication of having to ‘slide out’ the AED from the bespoke AED carrier that, to our experience, is an unusual way to open the device

Call-handler feedback: some volunteers struggled to open the case of the defib. Instructions could be included in the script so that they are able to open the defib quickly and effectively.

#1, participant: I couldn’t open it initially – looked like box with a lid; I could see how people would get flustered in real life.

#2, researcher: Looked briefly at AED before coming back to patient. Didn’t try to open AED until getting instruction to open by call-handler. Found no problems opening the AED in the end

#3, participant: There was no specific instruction from call-handler [Ed – about how to access/open AED]. A specific instruction would help: e.g. “open tab” “lift out”

#3, participant: AED – it was not obvious how to open using the mechanism. I didn’t open yellow tab at first.

#3, researcher: AED use - had to put phone down, needed both hands, took a while to work out how to open box

#4, participant: Once I got the device, I worked it out okay but wondered if others would be able to work it out. It was obvious what it was. “Not complicated”

#4, researcher: Opened AED without prompt.

#5, participant: I lost “vital” time trying to open AED. The call handler gave instructions but I had “no idea”.

#5, researcher: AED: Said “What the hell do I do with this” “I’ve never seen one of these before. Said “How do I open it”, several times.

#6, participant: The call-handler said “open” the AED; would be better to have the words “slide” or “pull here” on it in the relevant areas

#5, researcher: They really struggled to open the AED box.

#6, researcher: “How does it open” x 2 but call-handler “don’t have specific instructions.

#6, participant: it was simple enough to do. I had difficulty opening AED – it would have been nice to know it was a sliding door. I pulled out yellow tab – put it back in again as not sure if I should have or not

#6, researcher: Took a little while, but worked out how to open and use AED*.*

#8, participant: It was clear about how to open box – it was written on the box to pull yellow tab [Ed – actually, it wasn’t]

#10, participant: I was initially at a loss about what to do with the defibrillator – took a while to work out that had to pull the yellow lever to open the box. It was not at all obvious

- 1. Operating the AED

#1, participant: Defibrillator pads – it was unclear if the pads were universal / interchangeable.

#1, researcher: Participant said, “Are instructions on the defibrillator” Did a few compressions whilst trying to get defibrillator on. They placed the AED pads on top of jacket first, responded to “on bare chest” instructions, said “bare chest” to self out loud and corrected.

#1, researcher: Did not give shock (did not get pads off sticky backs). Discussed with call handler and came to agreement to re-start CPR. Restarted, scenario ended.

#1, participant: Defibrillator instructions – it didn't say straight on [Ed - bare] chest initially; I put it on the clothes, and the call-handler helped clarify need for putting on bare skin

#2, researcher: Said “Where’s the on button” and then quickly found it.

#8, researcher: AED use was incorrect: plugged in the connector before attaching pads, so it decided to shock before pads in position

#9, researcher: The sticky back from AED pads blew away as they opened it. They tried to grab it, initially but did not chase it

#10, researcher: AED. They retrieved it without difficulty. Pads were attached, but through clothes, and they didn’t respond / fix after “bare chest” prompt from the defibrillator, so, recorded as shock delivery, but inadequate / poor shock delivery

1. Bystander wellbeing

5.1 Bystander emotion

Call-handler feedback: I thought two of the volunteers were testing me rather than just the scripting for the defib due to them cutting me off or speaking over me. I can understand that whilst this may happen with members of the public, it wasn't necessary for this training. [Ed – this was NOT the case]

#2, participant [Ed - who couldn’t see drone approaching]: Drone – I was worried it might land ON me. Initially thought that might fly OVER me so I could see it. Being told, drone is on its way, it will land NEAR you and not fly OVER you might have helped

#5, researcher: Asked the call-handler, “I press every time I count?” (“1,2,3,4”) several times. They often spoke over the call-handler. They only started CPR once instructions had being given; then asked for CPR instructions to be repeated as they were actually doing it. Said: “Is there a speaker”, didn’t wait for answer, then “can you speak really loudly” please

#5, researcher: They asked clarification questions A LOT to the call-handler

#5, participant: I was worried about leaving the patient – I was told to continue compressions and how could I do that whilst leaving the patient. So, I ran as fast as I could

#5, researcher: Pressured speech throughout, unclear if this was real or trying to ‘act’ for the scenario. They often spoke over the call-handler. Shouting at several points. Said “I’m not sure I’m doing this right” Said, “Drone? Christ” to drone is coming instruction and “is an ambulance coming”

#6, participant: It felt good when I could see the drone – gave me confidence that the AED was on its way

#9, participant: Knowing that the drone was coming was reassuring – hearing the “whirring” of the drone is a bit like hearing the ambulance sirens and knowing help is on the way. Does it need a blue light or siren?

5.2 Advice and reassurance from the call-handler

General researcher observations: The drone has video / recording capability. Could this be accessed by call-handler – potential to assist whilst on-scene, without needing bystander action, and possible psychological support (e.g. if call-handler says, “I can see you”, “I can see you’ve got the AED” etc.

#4, researcher: They asked for a metronome tone from call-handler. Said “Are you still there” a few times to the call-handler, in response to periods of silence

#4, researcher: There was a lot of quiet/down time with call handler. The participant twice asked “are you still there” - the second time was when the drone winch was retracting after AED had landed

#4, researcher: In the hands a very competent individual, call-handler did very well in supporting role, not intervening unnecessarily.

#6, researcher: In response to the call-handler saying, “Shouldn’t be too long” the participant said “I can see it now”

#6, researcher: “Doing really well” “Are you ok” a few times from call-handler, unprompted, and “keep going”. Said “That’s brilliant, thank you”, when told drone on its way, with no clarification questions asked

#8, participant: The operator did not say to keep doing CPR*:* When I brought the package [Ed - AED] back from drone and was back at the patient’s side I asked “Do I need to continue CPR” and when I initially rung 999 – there was a delay to mentioning CPR

1. Participant perceptions of system efficacy

#2, participant: Excellent. I would be so relieved if I was in the middle of nowhere. It’s logical in that situation too – why do CPR if there is no AED available?

#3, participant: Could anybody do this? Not sure they could if they were older, not as fit – they couldn't do CPR for as long as in the scenario. Perhaps they’d get the energy from somewhere

#4, participant: Even if I had access to an AED nearby I would be tempted to stay with patient / do CPR and wait for the drone-delivered version

#5, participant: It would be quite a panic in a real-life situation. I can see how AED would work, but I can’t see how *I* could make it work [Ed - with how they performed / struggled to operate it]

#7, participant: It certainly seemed that the AED arrived swift – faster than ambulance in any case. The drone position seemed very accurate, it seemed as if it was right overhead

#7, participant: I couldn’t get up from knees after ending scenario – if had been life-and-death I would have crawled

#7, participant: The drone – you are reliant on this in a rural location, you are not able to get someone else to you there to help you.

#8, participant: Generally, it worked well

#9, participant: It was good, simple, straightforward; as long as weather conditions allow. I managed this despite the wind / drizzle. I think consumables might blow away in wind if doing outdoors in real life. Speed saves lives
